# The unvirtuous cycle of discrimination affecting people with hepatitis B: a multi-country qualitative assessment of key-informant perspectives

**DOI:** 10.1186/s12939-022-01677-6

**Published:** 2022-05-31

**Authors:** Catherine Freeland, Lindsay Mendola, Vivian Cheng, Chari Cohen, Jack Wallace

**Affiliations:** 1 Hepatitis B Foundation, 3805 Old Easton Rd, Doylestown, PA 18902 USA; 2 Temple University, 1801 N Broad St, Philadelphia, PA 19122 USA; 3 Arcadia University, 450 S Easton Rd, Glenside, PA 19038 USA; 4 Burnet Institute, 85 Commercial Rd, Melbourne, VIC 3004 Australia; 5 Center for Social Research in Health, UNSW, Sydney, NSW 2052 Australia; 6 La Trobe University, Bundoora, VIC 3086 Australia

**Keywords:** Hepatitis B virus, Stigma, Discrimination, Human rights, Marginalization, Health outcomes, Liver cancer

## Abstract

**Background:**

An estimated 296 million individuals live with chronic hepatitis B worldwide, most have not been diagnosed and remain at risk of liver disease and cancer. People with hepatitis B often face discrimination that denies them employment or education opportunities, results in unfair treatment at work or in school, limits their ability to emigrate to certain countries, and in some cases prohibits them from serving in the military. Discrimination specific to hepatitis B has not been widely documented within the literature. This study aims to investigate and describe hepatitis B related discrimination, document discrimination occurring around the globe, and provide initial recommendations for addressing discrimination using key informant interviews.

**Methods:**

Purposive and snowball sampling were used to identify potential key informants for qualitative interview. Key informants identified as community health leaders, public health scientists, doctors, and researchers, many of whom were also living with hepatitis B. Using a semi-structured guide, participants were asked to describe their experience and any challenges for people living with hepatitis B including marginalization and its’ consequences. A codebook was used to guide the organization of data for analysis, and all transcripts *N* = 17 were double coded.

**Results:**

The overarching themes identified from interviews demonstrate explicit experiences with discrimination of those directly affected, the psychological responses, and the negative health outcomes associated with the unvirtuous cycle of discrimination. All key informants reported on the substantial quality of life implications and often poorer health outcomes resulting from hepatitis B discrimination. Participants also identified the significant impact of hepatitis B discrimination occurring within a range of education-based services across several countries as well as military exclusion or removal if individuals are found to have hepatitis B.

**Conclusion:**

Our data demonstrate that hepatitis B discrimination has a significant impact. Discrimination can occur at various points in life from education, to seeking employment, to marriage, to restrictions on entry, travel and stay in other countries. This study demonstrates the impact of discrimination and the need for future research that can lead to policy change and protections for people living with and impacted by hepatitis B.

## Background

Hepatitis B is a virus that infects the liver and can lead to cirrhosis or liver cancer if unmanaged or untreated. In 2019, the World Health Organization (WHO) estimate 820,000 deaths worldwide attributed to chronic hepatitis B, mostly from cirrhosis and hepatocellular carcinoma (HCC, primary liver cancer) [1]. An estimated 296 million individuals live with chronic hepatitis B worldwide, most of whom have not been diagnosed and remain at risk of liver disease, including cancer [1, 2]. Clinical management, including regular monitoring and pharmaceutical treatment effectively reduces the risk of mortality resulting from the infection [3]. While most people are unaware of their hepatitis B infection, those that have been diagnosed often face significant social marginalization from stigma and discrimination as a result of their hepatitis B infection [1, 4–6].

People with hepatitis B often experience disease-related stigma [6, 7]. Stigma has been defined as a social process, experienced or anticipated, and which is characterized by exclusion, rejection, blame or devaluation resulting from experience, perception or reasonable anticipation of an adverse social judgement about a person or a group [8]. Stigma resulting from hepatitis B infection has contributed to discrimination, a reduction in quality of life, and difficulty accessing employment, education and immigration [7]. Within academic literature, limited research is available specifically describing hepatitis B related stigma [7] and the consequences of stigma, including discrimination.

People with hepatitis B often face discrimination that denies them employment or education opportunities, results in unfair treatment at work or in school, limits their ability to emigrate to certain countries, and in some cases prohibits them from serving in the military. Most research about discrimination has been conducted in China, or with people of Chinese ethnicity living in other countries. In 2010, China outlawed a requirement for hepatitis B testing for school admissions and employment applications [9, 10]. Despite this, the U.S. State Department’s 2015 human rights report noted that testing for hepatitis B remains common practice in employment screening in China [11]. It has been documented that people who test positive for hepatitis B are not hired and provided a reason other than their positive status [12]. There appears to be limited awareness of the anti-discrimination laws and workers’ rights in China as well as limited laws available to respond to hepatitis B specific discrimination [10]. Despite legal prohibitions in place in China, a survey done in Beijing, China in 2015 showed that 40% of participants with hepatitis B were required to undergo pre-employment testing, with 29% believing they lost employment opportunities due to their infection [13]. In the U.S., discrimination is experienced by healthcare students, causing denial of school admission or enrollment, restriction of clinical training or dismissal from an academic program [14].

While hepatitis B discrimination has largely been reported anecdotally within a global context, this study aims to investigate and describe hepatitis B related discrimination, document discrimination occurring around the globe, and provide initial recommendations for addressing discrimination using key informant interviews. For the purposes of this study, discrimination is described by modifying the HIV-specific discrimination definitions within the United Nations [15], and where discrimination specific to hepatitis B is defined as the unjust, unfair, or prejudicial treatment of a person on the grounds of their hepatitis B status. In other words, being treated differently from others in the community because of being infected with hepatitis B.

## Methods

### Data collection

Purposive and snowball sampling were used to identify potential key informants to interview. First, individuals were classified as key informants by investigators as being community health leaders, public health scientists (*N* = 3), doctors (*N* = 3), researchers (*N* = 1) and patient advocates (*N* = 17), many of whom were also living with hepatitis B (*N* = 8) and actively work in hepatitis B in their respective communities (*N* = 16). All were over 18 years old. All key informants could speak to hepatitis B- specific challenges related to discrimination from their professional research, personal, or community-based organization experiences most participants had lived experience related to hepatitis B and are known within the viral hepatitis community as champions and key leaders. Several participants started their own community-based organizations to help others after experiencing a personal diagnosis with hepatitis B (*N* = 8) and actively work on addressing community health needs in their respective communities (*N* = 17). Participants were approached via email by study staff (CF and LM), informed of the study goals and were requested an interview using the zoom platform. In total, 34 people were emailed with an interview request, some individuals did not agree to participate due to not having enough information to contribute (*N* = 3), their role being clinically focused (*N* = 2), while some were unreachable (*N* = 12).

Once an interview was scheduled by study staff (LM) the interviews were conducted by two trained facilitators (CF and LM) using a structured interview guide and using the Zoom platform. The structured interview guide was designed using literature on discrimination from other disease states, followed by expert review (Appendix). Participants were first asked to describe their work or experience as it relates to hepatitis B, describe challenges for people living with hepatitis B including marginalization in the communities the participant serves, if, why and how people with hepatitis B are marginalized, its’ consequences, and recommendations to addressing this marginalization.

At the end of each interview, participants were asked if there were other people we should speak to who had working knowledge of hepatitis B-specific discrimination or could speak to personal experiences with discrimination. Interviews took place between January 2021 to May 2021 and lasted 25 to 70 min in length. Each interview was conducted in the English language, electronically recorded and transcribed verbatim. Interviews were performed by study staff until saturation was reached and no new details were emerging from interviews related to hepatitis B discrimination.

### Data analysis

**Fig. 1:**
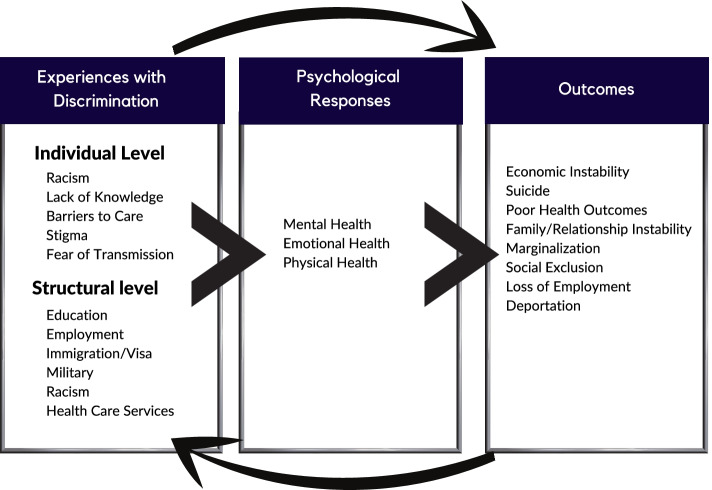
The cycle of discrimination experienced by those living with hepatitis B and outcomes as a result of this discrimination

A directed content analysis approach was used to create the codebook and guide the organization of data. Codes were developed by review of the literature (a priori) and through line-by-line reading of a subsample of queries [16]. Each code was given a specific definition to ensure coding accuracy and to improve intercoder reliability [17]. The codebook is provided as Fig. 1 and includes the themes and their frequencies of reference throughout the data analysis and coding process. All data transcripts (*N* = 17) were independently double coded by two members of the research team (LM, VW) to ensure coding accuracy. Inter-coder reliability (ICR) was assessed to identify coding discrepancies, and the analysis team met throughout the coding process to discuss and resolve discrepancies in coding. A kappa score of 0.80 or greater was considered good coding agreement. After coding was complete, the team reviewed the coding and organized findings into thematic categories. Data analysis used Nvivo version 12 (QRS International, Doncaster, Australia), a software program that facilitates organization of qualitative data. This study was approved by the Heartland Institutional Review Board with verbal consent obtained and recorded by all study participants prior to each interview. This study was performed in accordance with the ethical standards in the 1964 Declaration of Helsinki and its later amendments and comparable ethical standards.

## Results

Most study participants lived in hepatitis B endemic regions including Ethiopia, Nigeria (*N* = 2), Ghana, Uganda, Tanzania, Philippines (*N* = 2), Kiribati, China (*N* = 2), Mongolia, India, Turkey, and non-endemic regions including Australia, and the United States. Several overarching themes emerged related to experiences associated with discrimination and are depicted in Fig. 1. The themes demonstrate specific experiences with discrimination, the psychological response, and the health outcomes associated with an unvirtuous cycle that impacts people’s lives in various ways at various points in their life. Below we describe each element of the discrimination cycle: the causes or influences of discrimination, the experience and context of discrimination, and the outcomes of discrimination represented by quotes from all key informants. Strategies for addressing discrimination from the perspective of key informants is also included as recommendations towards addressing discrimination.

### Exposure to discrimination: psychological impact

All key informants reported on the substantial quality of life implications and often poorer health outcomes resulting from hepatitis B discrimination. For some, these had public health implications with several informants describing people with hepatitis B hesitating to get clinically managed for hepatitis B. One participant described the downstream health outcomes associated with discrimination, “*The side effect[s] and social impact really causes other people not coming out, not going to the hospital, not getting the proper treatment or testing*.” Similarly, one informant stated, “*because of the stigmas many people, they are not willing to go to hospital to test*.”

This study found psychological responses to discrimination including trauma, suicide, mental health challenges, depression, economic instability, and social isolation. One participant shared the broader impact of hepatitis B discrimination stating, *“[they] find out that they have hepatitis, so the problem now is since for example, you studied up to college and you are now to go to work and help your family then suddenly you are being denied [employment] not even once but twice or thrice so it really demoralizing.”*

Another participant explained challenges for women in relation to childbearing. In most countries it is recommended that women are mandatorily tested for hepatitis B during pregnancy to reduce hepatitis B mother to child transmission during delivery. The psychological and practical impact of this diagnosis on women can be substantial in a context where clinical guidelines are not followed: *“You hear people being forced to abort child, because during their pregnancy, there were found to be positive…these women and sometimes even by their own families they’re disowned.”*

Hepatitis B-related discrimination was identified to affect people with hepatitis B from a very young age: “*Even parents don’t like to send their children to school because again they believe the child is infected and that they might not even live long.”*

The impact of the social marginalization of people with hepatitis B was seen to cause significant mental health challenges, with one participant describing that, *“Because people are discriminating against them so some people can even kill, or [commit] suicide”,* while another noted that people with hepatitis B *“definitely feel very lonely because they got disease and they get rejected by their beloved, and their friends, and colleagues.”*

### Exposure to discrimination: individual impact

#### Stigma

Stigma, both self-stigma or stigma internalized by people as a result of external or public stigma, and public or external social stigma [18] were described as key factors contributing to discrimination.

Many of the informants, particularly those with hepatitis B described some of the social contexts that affected them daily. One key informant noted, *“One battle is inside the body that we are facing every day that effect[s] mental health or physical health and in society, we face discrimination, and you know disrespectful manners, we face stigma about the disease. For a hepatitis B positive patient, it's very difficult to survive in this world.”* Another person with hepatitis B noted that people with hepatitis B were deeply affected by concerns related to transmission “*people feel so much guilt about potentially transmitting the virus to your loved ones*.”

It was described how misunderstandings about the infection affect people’s lives in many ways, including their intimate, romantic and familial relationships. One informant shared, ‘[people think] *hepatitis is always a terminal illness, you will not live long so that is a barrier to most couples even getting married. They just feel people should not get involved with people like that*.” Other’s report being stigmatized because of their hepatitis B, one individual explained, “*when I go for a lecture in the community to educate about hepatitis B, most of the time I faced discrimination that people fear to touch and they hesitate to again, like [to have] lunch together or they hesitate to handshake*.”

Overall, a variety of factors including common misconceptions, lack of knowledge about transmission and low awareness specific to hepatitis B contribute to stigma that in turn can progress towards discrimination.

#### Lack of awareness

Overall lack of awareness of hepatitis B among the public is a significant contributor to discrimination according to several key informants. One participant shared, “*For me, I think the reason why hepatitis B patients are discriminated is because those who discriminate them in the first place, are not informed about hepatitis B*.”

Alternatively, one participant noted that increased knowledge would prevent discrimination within the workforce: “*if people knew [and] employers knew it needs blood contact to get hepatitis, they would not discriminate against people …but they say it is contagious, that it is airborne and it so …infectious so that’s why they discriminated against people even in jobs.”*

The impact of the lack of awareness about hepatitis B had a significant impact on the development of community based responses to the infection with one informant describing the fundraising challenges result from lack of community awareness and the unvirtuous cycle this produces: *“We have been trying as an organization to do (raise) awareness, but you as you might be aware, most of our funding is donor driven, and we don't have so many donors for hepatitis.”*

#### Misinformation

Overall participants overwhelmingly described how the significant level of misinformation specific to hepatitis B transmission, vaccination, and treatment was a primary cause of discrimination. Several participants noted the impact of misinformation in relation to transmission and vaccination:

*“People still view people with hepatitis as something that is contagious. People believe hepatitis as something that is airborne, people still believe that hepatitis can be spread in the workplace by sweat, [with] very low contact.”*

*“People still do not understand h[epatitis] B is being transmitted [and] they don't understand how the vaccine works. They don't understand how [hepatitis] is being transmitted. That's a problem.”*

This misinformation directly contributes to how people living with hepatitis B are treated.

*“Discrimination at home because … they believe that hepatitis is contagious [and] is airborne, [they think] you can get it by sweat just the ordinary sweat, or casual contact.”*

*“Everyone just feels that hepatitis a death sentence, so we don't want to get. From the marital perspective, no young lady wants to get engaged with the young man who has hepatitis B because he's going to die soon.”*

Others experience this discrimination and stigma in health care settings. One participant shared, *“And I even ran into a pediatrician who didn't believe that his patients [are] at risk because quote: ‘his patients never hung out drug dens’.”*

### Exposure to discrimination: structural impact

#### Education based discrimination

All individuals interviewed reported the significant impact of hepatitis B related discrimination occurring within a range of education-based services across several countries. Interviewees reported that these students, particularly people with hepatitis B who have immigrated to other countries, are, *“afraid of creating a stir so it [discrimination] doesn't get reported because their families are immigrants, they don't want to rock the boat they don't want media attention. They don't want to reduce their chances of getting into another school, you know if they come off as complainer.”*

Within healthcare schools (i.e. medical school, nursing school), limits on education for people with hepatitis B were described. One participant shared, *“I received a[n] email that a girl got a rejection in medical college just because she was a hepatitis B positive*.”, while another described, “*I've heard students who had to meet with their [advisor], they were told they couldn't rotate through certain rotations like surgery.”*

In the Philippines, one participant reported, “*We had a student who is a I think she was studying as a nurse, she was forced to drop out and transfer to another curriculum.”* Another participant described how, *“two students lost their medical acceptances just a couple weeks before matriculating: when they submitted their health forms and the letters written by the schools were just so mean they said, you are a threat to you yourself and others and you can't matriculate.”*

### Employment based discrimination

Employment based discrimination affecting people with hepatitis B was reported as occurring by all participants except those from Mongolia. Interviewees explained that people with hepatitis B were denied access to employment as a result of their hepatitis B status. One key stakeholder from India reported, “*In India, every day we hear about the denied employment, just because of the hepatitis B positive*.”

Some participants also reported employers conducting random and unnecessary workplace-based hepatitis B screening. In Ghana for example, an interviewee explained, “*I know about three guys that wrote me they work in a bank, and they did this random testing, and they were positive, and the result came out and the banks told them, they don't need their services anymore. Because of their hepatitis B status.”*

One informant from the Philippines noted consequences of pre-employment screening for hepatitis B. Key informants noted that in order to gain employment in many jobs, whether government or military, and even within some private industries screening for hepatitis B can take place and if someone tests positive for hepatitis B, they are denied employment despite their qualifications. This means that any regulation or law affecting employees cannot be used for redress. One individual described, *“Most of the cases is pre-employment. When it comes to pre-employment, since you're not still technically an employee it's very hard to complain. I mean you can go to the Department of Labor and complain, but some would say that because you're not an employee, management has the prerogative to decide whether to accept you, so you don't really have a choice.”*

The settings in which employment-based testing was broad with one participant noting restrictions occurring within the hospitality industry, “*When it comes to food-like company, producing food, hotel job, … people doing anything food …before you can get employment, you go for medical checkup. After medical checkup, if you are qualified, they will just call you… if you have any hepatitis B, they will disqualify.”*

Other forms of employment-based discrimination come from policing or military services in many countries with informants from Africa particularly, although not exclusively, noting this issue. A participant reported from Ghana, “*recruitment into the military when they do that test and they are positive, they will discriminate against them they don't get employed because of they are hep B positive.* Another participant shared their experience in Uganda, *“In the Uganda police force and Uganda people defense forces… when they are advertised for recruitment before you go for the training, they subject you to the tests. So, once they get you with hepatitis B that is enough to kick you out.”* Similar instances were described within Nigeria, “*Within Nigeria, we have people that are dropped from employment, especially some of the military organizations … when they believe you have hepatitis.”*

### Immigration/visa discrimination

Overseas work plays an important role in the economies of many of the countries represented by key informants and provides essential economic prospects and financial security for individuals where employment opportunities are limited and there are few job opportunities. However, having hepatitis B and seeking work abroad was reported by key informants as a significant challenge for many. People with hepatitis B are often restricted in obtaining visas to immigrate, work or train in other countries. One informant from Nigeria shared, *“[Getting a] work visa especially to the Asian countries does not happen, or they don't get work permits, because they have hepatitis.*”

Participants in Nigeria, Ghana and Uganda reported that, “*The Asia[n] countries if you're going there to work, they would deny you like Saudi Arabia, Kuwait, Qatar, they would deny you the visa. Or they would deport you.*” One individual shared, “*It mostly affects the patient, because one, you are qualified to work but because we are a hepatitis B patient, we have been disqualified.* Others reported similar experiences and for many people beyond countries within sub-Saharan Africa it was reported that *“In shipping or going abroad, sometimes it's [testing] also a requirement of course so we can't do anything about national law, but for other countries even.* Another individual described, *“those trying to go to the UAE they’re domestic helpers I think it depends on the employees if they will be accepted, because the way they do it is that the requirements are being processed in the Philippines if the employer would require these sets of test laboratory tests, I don't think the Agency can do anything about it.”*

### Pre-marital testing (reported in Uganda, Nigeria, Ghana, and Ethiopia)

Within many countries including, Uganda, Nigeria, India, Ghana, and Ethiopia, key informants reported customary pre-marital screening which includes infectious disease screening prior to engagement or marriage, which was reported to have a significant impact on people with hepatitis B and their communities. A participant in Ethiopia noted, “*They do the test, and they are positive, some of them don't marry the person because he's [or she is] positive.”* One of the participants from Nigeria described their own experience of being diagnosed with hepatitis B as a result of pre-marital testing, *“They wanted to test [for hepatitis as] part of premarital test. And when I discovered, I had this disease [hepatitis B], it was a tough time for me to discuss by status, it was a tough time because, for me, I felt I felt I was going to lose her. And the in laws are going to reject the marriage proposal.”*

Another participant from Ghana talked of the challenges associated with religiously-mandated pre-marital screening, and the impact of this testing on people with hepatitis B within this context, *“Mostly just because this is organized by religious organizations for intending couples to get the screenings. And then, some of them [organizations] lack this knowledge to know that if one partner is positive, then the other could be vaccinated so for some as soon as the one partner is positive, it becomes like an end to the relationship.”*

### Recommendations

Participants were asked to recommend what needed to occur to address hepatitis B related discrimination. A variety of suggestions were made including the development of comprehensive public education to address hepatitis B misinformation and lack of knowledge within the community, and among health care workers. Others reported the need for the systematic documentation of discrimination, and support for people living with and affected by hepatitis B with one participant noting, “*If we have more data to showcase and tell the world what is happening, I believe that will go a long way.”* To improve awareness a participant shared, “*For the patient you need a lot of support some of these community driven activities to drive to increase awareness, to get people tested. And then that will probably make change happen.*”

Most informants noted the importance of national policies to protect individuals living with hepatitis B from discrimination with one noting, *“First of all, we need a national policy.”* Another informant noted the experience of government response to the HIV epidemic and its success in developing systematic responses limiting testing, *“Policy, what we need is really like HIV, they have a very specific policy that pertains to testing, so the employer cannot force you to take a test like that, so what we want is similar to HIV.”* Another participant described the importance of partnership between government and people living with hepatitis B to effectively develop policy against discriminatory practices and explained, “*When we are proposing those policy changes, how would you expect their policies to change if you're not involving the people who are watching on the ground in the community.”*

## Discussion

While hepatitis B is a chronic manageable health condition, our data demonstrate that hepatitis B discrimination has a significant impact on the lives of those affected. Discrimination can occur in a cyclic nature at various points in an individual’s life from education, to seeking employment, to marriage, to restrictions on entry, travel and stay in other countries. Hepatitis B discrimination impacts mental and physical health of those experiencing it firsthand and limits overall wellbeing, quality of life, can lead to poor health outcomes and avoidance of testing and management. Within the context of HIV, and where discrimination related to HIV is well documented and has been for several years, hepatitis B related discrimination is occurring in health care settings, reducing access to health services or engaging in quality health care [19], and findings from this study demonstrate a parallel between the experience of HIV and hepatitis B within health care as key informants describe discrimination contributing to limiting access to health care services for clinical management, including diagnosis. Future research should continue to explore and document the impact of discrimination within the context of health care services and specifically hepatitis B.

Findings from this study illustrate sources of discrimination as a combination of lack of information, misinformation, and poor hepatitis B knowledge, which is consistent with previous research. Literature documents low knowledge related to transmission as a key contributor of discrimination within China specifically [9]. Within this study, key informants describe the limited understanding of hepatitis B transmission and prevention within populations specifically leading to stigma and discrimination [20–22]. Hepatitis B is primarily transmitted in countries with rigorous vaccination programs through unprotected sex or unsafe injecting and is understood and described within health systems as a sexually transmitted infection [23–25]. This framing can lead to further discrimination and stigma which was described by key informants as a significant challenge.

Our study identified many consequences of discrimination and the clear lack of knowledge associated with hepatitis B. An identified downstream impact is the promotion of false cures and treatments sought by people living with hepatitis B in many countries. Similar findings have been identified within literature, with a commentary on discrimination in China highlighting the impact of advertising of alleged cures and remedies that contribute to the deep antipathy towards people living with hepatitis B [9]. While many studies have demonstrated a clear gap in knowledge related to hepatitis B [7], efforts at every level should continue to work towards increasing knowledge and awareness of hepatitis B transmission, prevention, and diagnosis working to normalize conversation around hepatitis B to work towards reducing stigma and discrimination.

Within interviews, herbalists or traditional medicine was reported in several countries including Nigeria, Uganda, Ghana, and China and works to convince people living with hepatitis B that they can falsely be cured and remove the cause of their discrimination. Interviewees reported that in desperation, people living with hepatitis B are determined to try any means to remove the virus from their body. Key informants described that herbal or traditional medicine can provide a “promised solution” to remove hepatitis B, reduce their financial costs, provide access to a medication, and remove the thing that is causing their discrimination. That individuals are seeking false cures and traditional medicine highlights the lack of information and knowledge associated with the disease among those directly impacted by it. Additionally, the possible impact of herbal and traditional medicines in the context of hepatitis B discrimination needs further exploration.

Overall, hepatitis B discrimination was reported to affect health outcomes, mental health, contribute to economic and family instability, social isolation, suicide, loss of employment, and trauma. The impact of discrimination directly affects not only the individual but their families and relationships as seen with the described pre-marital testing in this study. It is important to further explore the context of pre-marital testing as authors anticipate it taking place in more than the countries explored within this study and of the consequences of this testing on people diagnosed with hepatitis B.

The educational and employment discrimination that people with hepatitis B face directly affects their economic contributions to their families and future opportunities. Unfortunately, in many reported instances, employment discrimination occurs through pre-employment or pre-admission screening when individuals have no authority to appeal or protest limiting their options and forcing individuals to accept what the employer or institution decides. Further, key informants describe many individuals in sub-Saharan Africa and the Western Pacific seek employment opportunities as domestic workers in the Middle East and parts of Asia. As people seek these opportunities, they are screened for hepatitis B and denied employment opportunities if positive hepatitis B test occurs. Denial of employment or education as a result of hepatitis B infection is unethical, and contradicts World Health Organization hepatitis B guidance [26]. There is no reason that people with hepatitis B should not be provided with the equal opportunities for employment, travel, and education and anything otherwise considered a human rights violation. As noted previously, most literature describing hepatitis B related stigma and discrimination comes from China or is related to people with Chinese ethnicity. Given the increasing global role of China and its economic support of many developing countries in Africa, Asia and the Pacific Islands, the social marginalization resulting from hepatitis B infection is being carried over with this support. It is normal in China for people with hepatitis B to be socially marginalized, with these norms affecting African students, and one participant from Africa noted “*For African nations to send young people to universities to study in China now part of that practice is screening them for HIV, syphilis and all major infectious diseases including hepatitis B and C. Those who are found to be positive, are essentially deported*.”

The same scenario commonly was reported to occur for health professionals across all countries with the denial of participating in clinical rotations or even attending school if found to be infected with hepatitis B during pre-enrollment screenings. The impact of discrimination in hepatitis B has been documented only within the context of China and medical students within U.S. with a similar consequence of negative emotional stress and anxiety [10, 14]. Even with protections and policy in place, refusal of admission within nursing and medical schools still is reported to occur [14].

There is a need to systematically document discrimination at local, country, regional and global levels and which should include the location, the causes of discrimination, where and how it is occurring at a local level. This documentation could be used to inform policy that ensures people with hepatitis B are provided protection and equal opportunity for employment, education, travel and immigration.

In some countries policies are in place to protect against discrimination specific to employment and individuals living with hepatitis B. In the United States, for example, individuals with hepatitis B fall under protections with the Americans with Disabilities Act and therefore have protections in place legally against discrimination [14]. This is not the case with militaries however, and even in the United States Military service, people living with hepatitis B are barred from deployment and have their careers put at risk due to infection. Within most countries, no transparent policies exist regarding hepatitis B despite reported discrimination occurring. In contrast to hepatitis B, most countries have policies in place against HIV discrimination, however, there is a need to make this policy broader and include diseases like hepatitis B to ensure discrimination is not tolerated. Additionally, the Global Health Sector Strategy on Viral Hepatitis emphasizes the need to address stigma and discrimination, but it is not written into the action plans of many countries. Future efforts should ensure that each country with elimination strategies include hepatitis B discrimination protections and policies.

In addition to implementing policy to prevent discrimination, there should be redress for individuals to address and report the discrimination that they experience. In China it has been documented despite a robust anti-discrimination policy, the discrimination is still widely occurring largely due to lack of knowledge of the existing policy at the local level [9]. Efforts should be made to ensure anti-discrimination policies trickle down to local and employer levels to ensure robust implementation.

Of the few published studies on hepatitis B discrimination within China, all recommended comprehensive informational campaigns to improve awareness regarding modes of hepatitis B transmission*,*and prevention through vaccination [9, 10]. The recommendations provided by our key informants is in line with the need for improved awareness in the general population.

Finally, those that have personal experiences related to discrimination should be at the forefront of the discussion and provided opportunities to share their lived experiences of how discrimination has directly impacted their lives and livelihood. To effectively address discrimination and stigma occurring related to hepatitis B those individuals who have firsthand knowledge of its consequences should be part of the solutions. Overall, discrimination specific to hepatitis B plays a considerable role in the lives of those directly affected and can occur at various points in an individual’s life. There is a need for more research, advocacy, and policy at all levels to effectively address discrimination, bring awareness to this issue, and work towards reducing its unjust consequences for those living with hepatitis B globally.

### Limitations

While this study provides valuable insight into hepatitis B discrimination and the consequences of that discrimination, there are important limitations that should be noted. First, while qualitative data collection methods provide rich, in-depth, experiential data, the data are limited in external validity (generalizability) and not representative of all experiences related to discrimination. While much of the sample identified as living with hepatitis B and had spent extensive time working in hepatitis B sectors, the sample size was small, only represented a small number of countries where hepatitis B is endemic, and future work should be done to gather and document additional data on hepatitis B discrimination globally. While issues of discrimination were noted by all informants, this is not to imply that hepatitis B related discrimination is an issue in all countries. Additionally, all interviews were conducted in English, limiting the global representativeness of the sample itself.

## Conclusion

Findings from our analysis demonstrate that hepatitis B discrimination has a significant impact on the lives of those infected. Discrimination can occur in a cyclic nature at various points in life from education, to seeking employment, to marriage, to restrictions on immigration entry, travel and stay in other countries. Hepatitis B discrimination has a substantial impact and affects an individual’s mental and physical health and limits overall wellbeing, quality of life, can lead to poor health outcomes and avoidance of testing and management. Future research should continue to examine the consequences of hepatitis B discrimination using systematic documentation of it happening on the ground at local, country, regional and global levels. Ultimately, policy change is needed to ensure people with hepatitis B are provided protection and equal opportunity for employment, education, travel, and immigration.

## Data Availability

Transcripts related to this study are available to corresponding author upon a reasonable request. Transcripts will not include any identifiable information if data is requested.

## Appendix

Table 1

**Table 1:** Hepatitis B discrimination qualitative interview codebook

Code Name	Code Description	Files	References
Alternative Medicine	Anytime a person is denied preventive care and resorts to using alternative medicine (ex: traditional medicine, herbalists, etc.)	5	19
Barriers		5	22
Cost Associated w Discrimination	Use when/if an individual describes economic loss or gain that is effected by discrimination	8	55
Descriptive Information	Use when an individual is referencing specific communities/countries/regions served. Or demographic information	15	44
Diagnosis	Use if the individual describes how they were diagnosed with hepatitis B and how it impacted their life related to discrimination. (This code might not be applicable to all participants, but many have described their diagnosis which led to them personally being discriminated against)	10	31
Education-Based Discrimination	Use when an individual describes challenges attending any form of schooling due to their hepatitis B status. Could mean withdrawn from an institution due to their hepatitis B status	10	28
Employment Based Discrimination	Use when individual describes discrimination that occurs within the setting of employment/occupation. This can include screening requirements pre or post-employment	13	109
Immigration Discrimination	Use when individual describes challenges associated with travel, visa access, or employment abroad in the context of living with hepatitis B	8	28
Impact of Discrimination	Use when individual describes the consequences, or effects that discrimination has on those living with hepatitis B or larger society as a whole. This can be either positive or negative impacts associated with discrimination. (Examples: mental health, suicide, isolation, job loss, other challenges)	14	181
Military Based Discrimination	Use when individual refers to discrimination in the context of the military	5	14
Other forms of Discrimination	Use when individual describes other forms of discrimination (Do not use if it relates to education, employment, immigration or military based discrimination)	10	45
Overcoming Discrimination	Use when individual describes current methods of overcoming stigma or discrimination or suggests strategies to overcome discrimination or stigma associated with hepatitis B	13	247
Pre-marital testing	Use when the individual describes getting tested for hepatitis as part of the process before marriage	5	16
Reason for Discrimination	Use when the individual describes their experience or understanding related to why individuals are being discriminated against (Examples (from interviews so far) include lack of knowledge related to hepatitis B prevention and transmission)	14	147
Stigma	Use when the person describes or mentions being treated differently due to their hepatitis B status	13	123

## Abbreviations

WHO: World health Organization
HCC: Hepatocellular Carcinoma
HIV: Human Immunodeficiency Virus
